# The effect of infection order of porcine circovirus type 2 and porcine reproductive and respiratory syndrome virus on dually infected swine alveolar macrophages

**DOI:** 10.1186/1746-6148-8-174

**Published:** 2012-09-25

**Authors:** Yi-Chieh Tsai, Hui-Wen Chang, Chian-Ren Jeng, Tsang-Long Lin, Chun-Ming Lin, Cho-Hua Wan, Victor Fei Pang

**Affiliations:** 1 Graduate Institute of Veterinary Medicine, School of Veterinary Medicine, National Taiwan University, Taipei, 106, Taiwan, Republic of China; 2 Graduate Institute of Molecular and Comparative Pathobiology, School of Veterinary Medicine, National Taiwan University, Taipei, 106, Taiwan, Republic of China; 3 Department of Veterinary Pathobiology, Purdue University, West Lafayette, IN, 47907-2065, USA

**Keywords:** Porcine circovirus type 2, Porcine reproductive and respiratory syndrome virus, Alveolar macrophages, Porcine respiratory disease complex

## Abstract

**Background:**

Concurrent infection with porcine circovirus type 2 (PCV2) and porcine reproductive and respiratory syndrome virus (PRRSV) is known as one of the major causes for porcine respiratory disease complex (PRDC). Dual infection with PCV2 and PRRSV is consistently to have more severe clinical presentations and pulmonary lesions than infection with PCV2 alone or PRRSV alone. However, it is not known if dual infections with PCV2 and PRRSV in different infection order may lead to different clinical symptoms in the host. To mimic the possible field conditions, swine alveolar macrophages (AMs) were inoculated with PCV2 and PRRSV *in vitro* simultaneously or with one virus 18 h earlier than the other. The cell viability, cytopathic effects, antigen-containing rates, phagocytotic and microbial killing capabilities, cytokine profiles (IL-8, TNF-α, and IFN-α) and FasL transcripts were determined, analyzed, and compared to prove the hypothesis.

**Results:**

A marked reduction in PRRSV antigen-containing rate, cytopathic effect, and TNF-α expression level was revealed in AMs inoculated with PCV2 and PRRSV simultaneously and in AMs inoculated with PCV2 first then PRRSV 18 h later, but not in AMs inoculated with PRRSV first then PCV2 18 h later. Transient decrease in phagocytosis but constant reduction in microbicidal capability in AMs in the group inoculated with PCV2 alone and constant decrease in phagocytosis and microbicidal capability in AMs in all PRRSV-inoculated groups were noted. The levels of IL-8, TNF-α, IFN-α, and FasL transcripts in AMs in all groups with dual inoculation of PCV2 and PRRSV were significantly increased regardless of the infection orders as compared with infection by PCV2 alone or PRRSV alone.

**Conclusions:**

Swine AMs infected with PCV2 first then PRRSV later or infected with PCV2 and PRRSV simultaneously displayed marked reduction in PRRSV antigen-containing rate, cytopathic effect, and TNF-α expression level. The different inoculation orders of PCV2 and PRRSV in AMs leading to different results in viral antigen positivity, cytopathology, and cytokine profile may explain, at least partially, the underlying mechanism of the enhanced pulmonary lesions in PRDC exerted by dual infection with PCV2 and PRRSV and the variable clinical manifestations of PRDC-affected pigs in the field.

## Background

Porcine respiratory disease complex (PRDC) is one of the major problems to the swine industry worldwide and arguably the most important swine health concern for the swine producers today. During the past 30 years, swine production has been intensified with larger herd sizes and confinement rearing, contributing to the increased incidence and complexity of the respiratory diseases. Porcine respiratory disease complex often occurs in pigs around 6 to 20 weeks of age, especially in large pig farms with continuous production system [1]. It is characterized clinically by slow growth, decreased feed efficiency, anorexia, lethargy, fever, cough, and difficult breathing [1,2]. A multifactorial complex of swine respiratory pathogens has been reported to play a role or roles in PRDC, including bacteria and viruses, which are complicated by management and environmental factors [1,3]. However, the pathogens involved vary significantly among farms and production sites. The viral agents that have been isolated from PRDC are porcine reproductive and respiratory syndrome virus (PRRSV), porcine circovirus type 2 (PCV2), swine influenza virus (SIV), pseudorabies virus (PRV), porcine respiratory coronavirus (PRCV), and recently Torque Teno viruses (TTV) [1-7]. The bacterial pathogens involved in PRDC include *Mycoplasma hyopneumoniae*, *Pasteurella multocida*, *Actinobacillus pleuropneumonia*, *Streptococcus suis*, *Haemophilus parasuis*, and *Salmonella enterica serotype Chloeraesuis*[1,3,7]. Procine circovirus type 2, PRRSV, *P. multocida*, and *M. hyopneumoniae* are the most important PRDC-inducing viral and bacterial pathogens, respectively, in Taiwanese swine industry [7].

Porcine circovirus type 2 and PRRSV have been suggested to be two of the important etiological factors for PRDC, and pigs with dual infections of PCV2 and PRRSV, however, consistently have more severe clinical symptoms and interstitial pneumonia [3,4,8-10]. Swine alveolar macrophages (AMs) co-inoculated with PCV2 and PRRSV have been shown to have significantly higher expression levels of Fas ligand and Fas than those inoculated with PRRSV alone [11]. In addition, co-infection of PCV2 and PRRSV in piglets synergistically has been found to suppress the mRNA expression profiles of T helper (Th) 1- and Th2-type cytokines in the peripheral blood mononuclear cells (PBMCs) [12]. Furthermore, dual infection of PCV2 and PRRSV in pigs with a PCV2 mutant that has the mutation at the interferon-stimulated response element (ISRE)-like element could exacerbate the pathological lesions and increase the PCV2 viral DNA load in the tissues [13]. These findings indicate that the interactions of PCV2 and PRRSV are critical to the pathogenesis of PRDC.

Pulmonary alveolar and/or intravascular macrophages are known as the major target cells for both PCV2 and PRRSV in the lungs [14-17]. We have previously used *in vitro* approaches to study the effect of infection with PCV2 alone [15] or PRRSV alone [18] on the functional changes of swine AMs; it was found that either PCV2 alone or PRRSV alone could cause significant reduction in the microbicidal capability and induce changes in expression levels of cytokine and chemokine, which may explain partially the pathologic changes in the infected pig lungs. In a co-infection study with PCV2 and PRRSV, instead of observing an enhanced effect, PCV2 reduced PRRSV replication and PRRSV-associated cytopathy by inducing IFN-α production in swine AMs [14]. Such findings, however, do not reflect and explain the enhanced clinical disease observed in PRRSV and PCV2 dually infected cases in the field. Therefore, the effects of dual PCV2 and PRRSV infection on the functions of swine AMs need further elucidated.

In a pig farm infected with PCV2 and PRRSV, it is conceivable that individual pigs may be attacked by both viruses in different sequence or order. The objective of the present study was to determine if different infection orders of PCV2 and PRRSV would result in different consequences in the functions of swine AMs by the *in vitro* inoculation of swine AMs with PCV2 and/or PRRSV.

## Methods

### Experimental animals and viruses

The animal use and care protocol was reviewed and approved by the Institutional Animal Care and Use Committee (IACUC) of National Taiwan University (NTU). Twelve, 5 to 6 weeks old, specific pathogen free (SPF) pigs were obtained from the Animal Technology Institute Taiwan; they were certified to be free of classical swine fever virus, foot and mouth disease virus, Aujeszky’s disease virus, *Toxoplasma gondii*, *P. multocida*, *Bordetella bronchiseptica*, *M. hyopneumoniae*, *A. pleuropneumoniae*, and *Brachyspira hyodysenteriae.* The SPF pigs were also tested negative for PRRSV, PCV1, and PCV2 antibodies and nucleic acids by immunofluorescent antibody assay (IFA) or real time PCR and used for the collection of AMs. These pigs were kept in the isolated laboratory animal facility with air conditioning system. Animal euthanasia was conducted in accordance with the Report of the AVMA Panel on Euthanasia and under the supervision of IACUC of NTU.

The stock of PRRSV used in the study was the 8th passage of a Taiwan field isolate, PRRSV tw91, at a titer of 10^7^ TCID_50_/ml, which was prepared and titrated on MARC-145 by cytopathic effect (CPE) [18]. The PCV2 used in the study was a field isolate by using pooled spleen and lymph nodes from a PMWS-affected pig with a PCV1/PCV2-free PK-15 cell line; the virus was sequenced as PCV2b subtype and the stock was prepared as previously described at a titer of 5 x 10^6^ TCID_50_/ml [14].

### Preparation of AMs

The bronchoalveolar lavage and collection of AMs were performed as described previously [14]. The AMs were adjusted to 5 x 10^5^ cells/ml in RPMI-1640 medium (Gibco Laboratories, Grand Island, NY, USA) supplemented with 10% heat-inactivated fetal bovine serum (FBS) (Hyclone, Laboratories, Logan, UT) and 1% 100x antibiotic-antimycotic solution (Gibco), containing 10,000 units of penicillin, 10,000 μg of streptomycin, and 25 μg of amphotericin B/ml in 0.85% saline, (RPMI-C). Three milliliters/flask or 0.5 ml/well of AMs were placed in Teflon flasks (Nalgene Company, Rochester, USA) or 24-well culture plates (Costar, Cambridge, MA, USA) with or without coverslips (Assistent, Nürtingen, Germany), respectively, and immediately exposed to one or both viruses or equal volume of RPMI-C. The flasks and culture plates were incubated at 37 in 5% CO_2_ for various time intervals as indicated below. The AMs were subsequently evaluated for antigen-positive rate, survival rate, TUNEL-positive rate, phagocytosis, microbial killing capacity, cytokine production, and Fas and FasL mRNA expression.

### Experimental design

Six groups of AMs were used, including AMs inoculated with PCV2 alone (PCV2), AMs inoculated with PRRSV alone (PRRSV), AMs inoculated with PCV2 first then inoculated with PRRSV 18 h later (PCV2/PRRSV), AMs inoculated with PRRSV first then inoculated with PCV2 18 h later (PRRSV/PCV2), AMs co-inoculated with PCV2 and PRRSV simultaneously (PCV2-PRRSV), and AMs inoculated with an equal volume of RPMI-C (Mock). The multiplicity of infection (m.o.i.) for PCV2 and PRRSV was 0.1 each, respectively. At 18, 36, 54, 72, 90, and 108 h post inoculation (HPI) with the first virus, AMs or supernatants from all of the treatment groups were collected and used for assays of antigen-positive rate, survival rate, TUNEL-positive rate, phagocytosis, microbial killing capacity, and cytokine production. For the measurement of mRNA expression of Fas and FasL, AMs were collected at 42 HPI after the removal of culture supernatant.

### Antigen-positive rate

The PCV2 antigens and nucleocapsid protein of PRRSV were detected by IFA with specific antibodies as described previously [14]. The rates of positivity were determined by counting 200 cells out of 10 randomly selected fields at 400x magnification on a fluorescent microscope (Optiphoto II, Nikon, Tokyo, Japan).

### Survival rate

The cytocidal effect of PCV2 and/or PRRSV on AMs was determined using the trypan blue dye exclusion assay [14]. The numbers of trypan blue-positive and trypan blue-negative cells were counted on a hemocytometer by light microscopy as described previously [18]. The survival rate (SR) was recorded as [(number of trypan blue-negative cells/total number of trypan blue-positive and trypan blue-negative cells) x 100%]. Data of SR were expressed as the level different from that of the Mock: [(SR value of PCV2- and/or PRRSV-inoculated group) - (SR value of Mock group)].

### TUNEL-positive rate

The terminal deoxynucleotidyl transferase (TdT)-mediated dUTP nick end-labeling (TUNEL) assay was performed according to the manufacturer’s instructions (*in situ* cell death detection kit, fluorescein; Boehringer Mannheim, Mannheim, Germany) and counterstained with 1% Hoechst 33258 (Sigma) as described previously [14]. The percentage of TUNEL-positive cells was determined by counting 200 cells out of 10 randomly selected fields at 400x on a fluorescent microscope (Optiphoto II, Nikon). The TUNEL-positive rate (TR) was recorded as [(number of TUNEL-positive cells/200 Hoechst-positive cells) x 100%]. Data of TR were expressed as the level different from that of the Mock: [(TR value of PCV2 and/or PRRSV-inoculated group) - (TR value of Mock group)].

### Phagocytosis and microbial killing assays

The phagocytosis and microbial killing assays were carried out as those described previously [15] by using *Candida albicans* as the target. Briefly, monolayers of AMs were incubated with spores of *C. albicans*, opsonised with pooled swine hyperimmune serum, in an AMs to yeast ratio of 1:10 for 60 min. Following PBS wash to remove non-phagocytized yeasts, the monolayers of AMs were stained with acridine orange (AO) (Sigma) and counterstained with crystal violet (Sigma). Under a fluorescent microscope (Optiphoto II, Nikon), the dead yeasts displayed an orange fluorescence and the viable ones had a green fluorescence. The number of AMs containing 1 or more yeasts in 200 randomly selected viable cells and among which the number of AMs containing killed yeasts were counted manually. The phagocytotic rate (PR) and microbicidal rate (KR) were recorded as [(number of AMs containing 1 or more yeasts in randomly selected viable cells/total number of viable cells) x 100%] and [(number of AMs containing killed yeasts in randomly selected viable cells/total number of viable cells) x 100%]. They were further expressed as the level different from that of the Mock: [(PR value of PCV2- and/or PRRSV-inoculated group) - (PR value of Mock group)] and [(KR value of PCV2- and/or PRRSV-inoculated group) - (KR value of Mock group)], respectively.

### Interleukin 8 (IL-8) and tumor necrosis factor (TNF)-α titration

The protein levels of IL-8 and TNF-α in the supernatants collected at each time point were determined using the commercial ELISA kits (Biosource, Camarillo, California, USA) according to the manufacturer’s instruction. The plates were read on an ELISA reader (Bio-Tek Instruments, Vermont, USA) at 550 nm. Data were expressed as mean concentration at pg/ml, where they were obtained by converting the OD value of each sample to the corresponding concentration based on the standard curve obtained from the 10-fold serial dilution of each recombinant porcine cytokine with known concentration. They were further expressed as the level different from that of the Mock: [(value of PCV2- and/or PRRSV-inoculated group) - (value of Mock group)].

### Interferon (IFN)-α bioassay

The IFN-α bioassay was performed as described previously by using Madin-Darby bovine kidney (MDBK) cells and vesicular stomatitis virus (VSV) [14]. Recombinant porcine IFN-α (Chemicon, Temecula, California, USA) was used as a standard. Data were recorded as mean unit (U)/ml, where 1 U of IFN-α activity was defined as the reciprocal of the dilution producing 50% inhibition of cytopathic effect (CPE). They were further expressed as the level different from that of the Mock: [(value of PCV2- and/or PRRSV-inoculated group) - (value of Mock group)].

### Semiquantitative analysis of Fas and FasL transcripts by RT-PCR

Total RNA was extracted from AMs using TRIzol reagent (Life Technologies, Paisley, Scotland, UK), reverse-transcribed to cDNA, and amplified by PCR with the following primers: porcine Fas (sense: 5^′^-GCA GGA TCC AGA TCT AAT CTA CAC-3^′^, antisense: 5^′^-CTA GGC AGG TTG TTT AGA GGC AGT-3^′^) [19], porcine FasL (sense: 5^′^-AAT GGG AAG ACA CCT ATG GAA-3^′^, antisense: 5^′^-CTT AGA GCT TAT ATA AGC CGA AAA ACG TC-3^′^) [20], and a porcine internal control GAPDH (sense: 5^′^-ACC TCC ACT ACA TGG TCT ACA TGT TC-3^′^, antisense: 5^′^-CAT TGA TGA CAA GCT TCC CAT TC-3^′^). Amplifications were performed with a thermocycler (MJ Research, Watertown, MA) using 30 cycles (95°C for 45 s, 62°C for 45 s, 72°C for 1 min) for Fas; 33 cycles (94°C for 40 s, 55°C for 40 s, 72°C for 1 min) and additional extension at 72°C for 5 min at the end of amplification for FasL; and 40 cycles (95°C for 15 s, 60°C for 1 min, 72°C for 1 min) for GAPDH. The PCR products were separated on a 2% Tris/Borate/EDTA (TBE) agarose gel by electrophoresis and stained with ethidium bromide (Sigma). The gels were photographed and analyzed using the ChemiDoc^™^ XRS (Bio-Rad, Segrate, Milan, Italy). The intensity of Fas or FasL was normalized to that of the GAPDH. Data were expressed as the level different from that of the Mock: [(value of PCV2- and/or PRRSV-inoculated group) - (value of Mock group)].

### Statistical analysis

The means presented in figures and used in statistical analyses represent at least three independent trials per main experiment run concurrently. The effects of the different treatments on antigen-positive rate, survival rate, TUNEL-positive rate, phagocytotic and microbial killing capabilities were analyzed by generalized linear models with the use of PROC GENMOD and binomial statement in Statistical Analysis System (Statistical Analysis System; SAS for windows 6.12; SAS Institute Inc., Cary, NC, USA). The *P* values were then adjusted by using Bonferroni test in the MULTTEST procedure in SAS. The data of cytokine expression levels and levels of Fas/FasL transcripts were analyzed by analysis of variance (ANOVA) followed by Duncan's multiple-range test carried out by SAS procedures. A *P* value of less than 0.05 was considered significant.

## Results

### Antigen-positive rate of PCV2- and/or PRRSV-inoculated swine AMs

**Figure 1:**
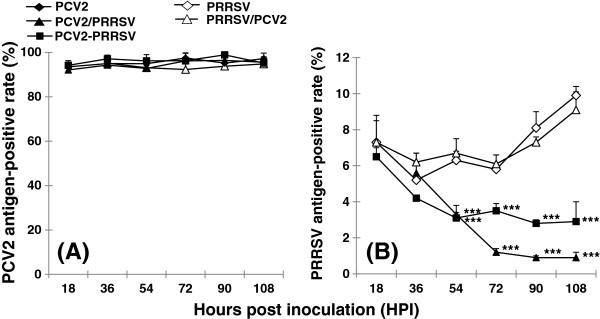
**Changes in PCV2 and PRRSV antigen-positive rate in swine alveolar macrophages (AMs).** Changes in (**A**) PCV2 and (**B**) PRRSV antigen-positive rate in PCV2- and/or PRRSV-inoculated swine alveolar macrophages (AMs) were determined by indirect immunofluorescence assay. Data are expressed as percentage and shown as mean ± SD of three independent experiments. Solid diamond: inoculation with PCV2 alone (PCV2); open diamond: inoculation with PRRSV alone (PRRSV); solid square: inoculation with PCV2 and PRRSV simultaneously (PCV2-PRRSV); solid triangle: inoculation with PCV2 for 18 h first then PRRSV later (PCV2/PRRSV); open triangle: inoculation with PRRSV for 18 h first then PCV2 later (PRRSV/PCV2). *** ^(*P* < 0.001)^ The values are significantly different from PRRSV or PRRSV/PCV2 at the same h post PRRSV inoculation.

During 18 to 108 HPI, a steady positive rate of pin-like intracytoplasmic signals for PCV2 antigens, ranging from 91.7 ± 2.3 to 96.3 ± 0.5%, was detected in all groups receiving PCV2 (Figure 1A); a low but constant intracytoplasmic PRRSV antigen-positive rate, about 5.2 ± 1.0 to 9.9 ± 0.2% and 6.1 ± 0.5 to 9.1 ± 1.3%, was noted in the groups of PRRSV and PRRSV/PCV2, respectively (Figure 1B). No intranuclear PCV2 or PRRSV antigens were observed during the experimental period. In the groups of PCV2/PRRSV and PCV2-PRRSV, however, the PRRSV antigen-positive rates were 5.6 ± 1.1% and 4.2 ± 0.1% at 36 HPI, but it gradually reduced to 1.2 ± 0.2 % and 3.5 ± 0.4% at 72 HPI, then further dropped to 0.9 ± 0.3% and 2.9 ± 1.1% by 108 HPI, respectively. They were constantly and significantly lower (*P* < 0.041) than those of PRRSV and PRRSV/PCV2 groups during 54 to 108 HPI (Figure 1B). Neither PCV2 nor PRRSV antigens were detected in AMs from the pigs in the Mock group.

### Survival rate of PCV2- and/or PRRSV-inoculated swine AMs

**Figure 2:**
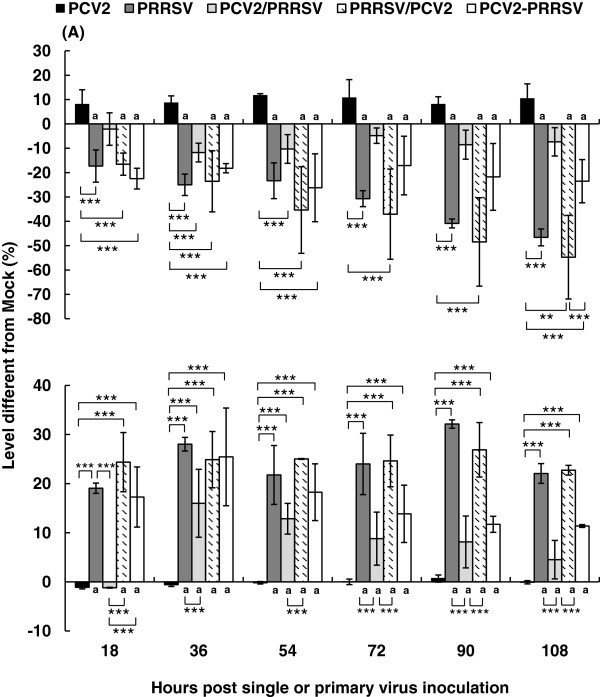
**Changes in survival rate and apoptotic rate of swine alveolar macrophages (AMs).** Changes in (**A**) survival rate and (**B**) apoptotic rate in PCV2- and/or PRRSV-inoculated swine alveolar macrophages (AMs) were determined by trypan blue dye exclusion assay and TUNEL assay, respectively. Data are expressed as the level different from that of the mock-inoculated AMs (Mock) and shown as mean ± SD of three independent experiments. PCV2: AMs inoculated with PCV2 alone; PRRSV: AMs inoculated with PRRSV alone; PCV2/PRRSV: AMs inoculated with PCV2 first then inoculated with PRRSV 18 h later; PRRSV/PCV2: AMs inoculated with PRRSV first then inoculated with PCV2 18 h later; PCV2-PRRSV: AMs co-inoculated with PCV2 and PRRSV simultaneously. ** ^(*P* < 0.01),^ *** ^(*P* < 0.001)^The difference between the two treatment groups at the same h post inoculation (HPI) with the first virus is statistically significant. ^a^Values are significantly different from the Mock at the same h post inoculation (HPI) with the first virus.

Before virus inoculation, the SR of AMs was 97.5 ± 0.7%. During 18 to 108 HPI, it became 80.1 ± 1.9 to 83.7 ± 1.5% and 88.8 ± 0.9 to 93.5 ± 2.5% in the Mock and PCV2 groups, respectively. The SRs of AMs in the PCV2 group were 8.1 ± 6.0% to 11.7 ± 0.7% higher than those of the Mock group, however, the differences were not statistically significant (*P* > 0.269). On the contrary, the SRs of all PRRSV-inoculated groups, except for the PCV2/PRRSV group, decreased after PRRSV inoculation and were significantly lower (*P* < 0.001) than those of the Mock and PCV2 groups throughout the experimental period. The SRs reduced in a time-dependent manner in the PRRSV and PRRSV/PCV2 groups; they were 66.0 ± 3.4% and 66.8 ± 1.3% at 18 HPI and dropped to 35.1 ± 3.2% and 27.0 ± 16.5% at 108 HPI, respectively, with no significant difference (*P* > 0.275) between each other at any time points. The SRs of AMs in the PCV2/PRRSV groups were 80.1 ± 4.9% at 18 HPI and slightly reduced from 76.3 ± 4.5% to 68.3 ± 5.7% after PRRSV inoculation during 36 to 108 HPI with no significant differences from those of the Mock group. However, they were significantly lower (*P* < 0.001) than those of the PCV2 group at 36 HPI, but significantly higher (*P* < 0.02) than those of the PRRSV and PRRSV/PCV2 groups at 18 HPI, 72 to 108 HPI and during 72 to 108 HPI, respectively. In PCV2-PRRSV group, the SRs of AMs were 55.6 ± 12.1% to 64.0 ± 14.0% during the experimental period and were significantly higher (*P* < 0.001) than those of the PRRSV and PRRSV/PCV2 groups at 108 HPI (Figure 2A).

### TUNEL-positive rate of PCV2- and/or PRRSV-inoculated swine AMs

The TRs of AMs were very low in both Mock and PCV2 groups throughout the experimental period. They were 1.4 ± 0.3% to 2.7 ± 0.1% for the Mock group and 1.4 ± 0.3 to 2.3 ± 0.8% for the PCV2 group. However, a significant increase (*P* < 0.037) in TRs was seen after the addition of PRRSV in all PRRSV-inoculated groups as compared with those of the Mock and PCV2 groups. The TRs of AMs in the PRRSV and PRRSV/PCV2 groups were 21.8 ± 1.1% to 33.9 ± 0.7% and 25.0 ± 1.0 to 28.6 ± 5.5%, respectively; they were consistently higher than those of the Mock group but there were no significant differences (*P* > 0.133) between the two groups during the experimental period. The TRs of AMs in the PCV2/PRRSV group increased to 18.1 ± 6.9% at 36 HPI after PRRSV inoculation, but they gradually reduced to 6.8 ± 3.1% by 108 HPI. The TRs of AMs in the PCV2-PRRSV group were 17.8 ± 2.5% at 18 HPI, raised to 26.7 ± 3.1% at 36 HPI, then gradually reduced to 12.3 ± 1.7% by 108 HPI. They were significantly lower (*P* < 0.001) than those of the PRRSV and PRRSV/PCV2 groups at 90 to 108 HPI (Figure 2B).

### Phagocytosis and microbicidal capacity of PCV2- and/or PRRSV-inoculated swine AMs

**Figure 3:**
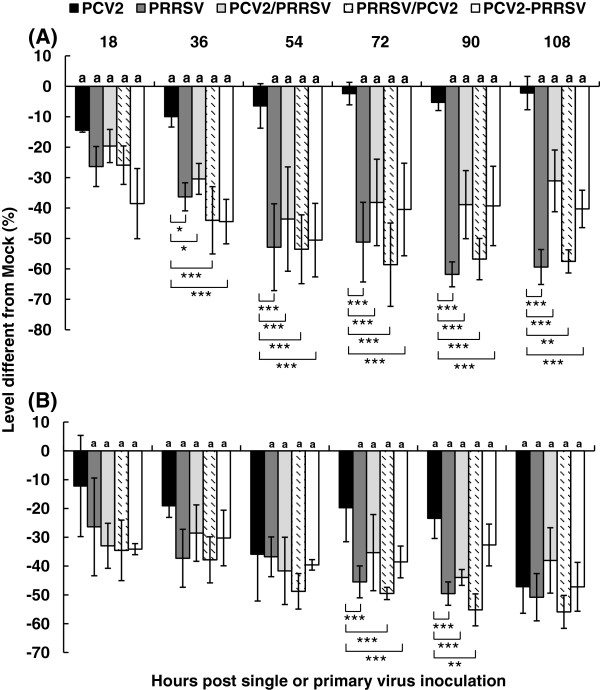
**Changes in phagocytotic and microbial killing capabilities of swine alveolar macrophages (AMs).** Changes in (**A**) phagocytotic and (**B**) microbial killing capabilities in PCV2- and/or PRRSV-inoculated swine alveolar macrophages (AMs) were determined by using *Candida albicans* as the target. Following 60 min of incubation and staining with acridine orange, the percentages of viable AMs with engulfed yeasts and killed yeasts were determined. Data are expressed as the level different from that of the mock-inoculated AMs (Mock) and shown as mean ± SD of three independent experiments. PCV2: AMs inoculated with PCV2 alone; PRRSV: AMs inoculated with PRRSV alone; PCV2/PRRSV: AMs inoculated with PCV2 first then inoculated with PRRSV 18 h later; PRRSV/PCV2: AMs inoculated with PRRSV first then inoculated with PCV2 18 h later; PCV2-PRRSV: AMs co-inoculated with PCV2 and PRRSV simultaneously. * ^(*P* < 0.05),^ ** ^(*P* < 0.01),^ *** ^(*P* < 0.001)^The difference between the two treatment groups at the same h post inoculation (HPI) with the first virus is statistically significant. ^a^Values are significantly different from the Mock at the same h post inoculation (HPI) with the first virus.

As far as the phagocytosis rate (PR) was concerned (Figure 3A), 88.1 ± 0.6 to 91.0 ± 5.5% of AMs in the Mock group were able to engulf one or more *C. albicans* during 18 to 108 HPI. The PRs of AMs in the PCV2 group were constantly lower than those of the Mock group with differences ranging from 2.2 ± 5.5% to 14.4 ± 0.6% throughout the experimental period and were significantly different from those of the Mock group during 18–36 HPI (*P* < 0.001). The PRs of AMs from all PRRSV-inoculated groups were consistently and significantly lower (*P* < 0.026) than those of the Mock and PCV2 groups after PRRSV inoculation during 36–108 HPI. As for the PRs of AMs in the PCV2/PRRSV and PCV2-PRRSV groups, the average PRs were similar between the two groups throughout the study but they were significantly higher (*P* < 0.015) than those of the PRRSV group during 90 to 108 HPI.

With regard to the microbicidal capacity (KR) (Figure 3B), 73.3 ± 10.5 to 76.2 ± 5.9% of AMs in the Mock group engulfed one or more *C. albicans* during the experimental period. The KRs of AMs from all PCV2- and/or PRRSV-inoculated groups (except for the PCV2 group at 18 HPI) were consistently and significantly lower (*P* < 0.001) than those of the Mock group during the experimental period with differences ranging from 26.4 ± 10.1% to 55.9 ± 5.7%. No statistical differences (P > 0.137) in the KRs of AMs were seen among all PRRSV-inoculated groups although there was a relatively lower KR in the PRRSV/PCV2 group than in the PCV2/PRRSV, PCV2-PRRSV, and PRRSV groups.

### Interleukin 8 (IL-8), tumor necrosis factor (TNF)-α, and interferon (IFN)-α levels in the supernatants of PCV2- and/or PRRSV-inoculated swine AMs

**Figure 4:**
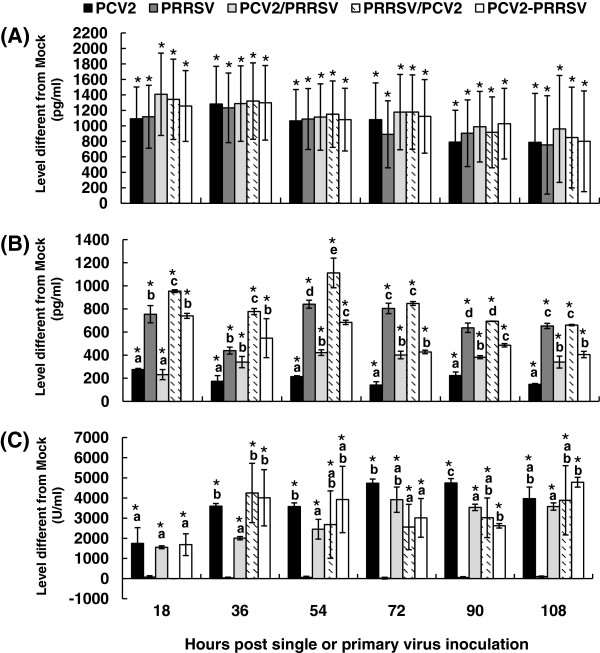
**Changes in the levels of IL-8, TNF-α, and IFN-α production in the supernatants of swine alveolar macrophages (AMs).** Changes in the levels of IL-8 (**A**), TNF-α (**B**), and IFN-α (**C**) produced in PCV2- and/or PRRSV-inoculated swine alveolar macrophages (AMs) were expressed as the level different from that of mock-inoculated AMs (Mock) and shown as mean ± SD of three independent experiments. PCV2: AMs inoculated with PCV2 alone; PRRSV: AMs inoculated with PRRSV alone; PCV2/PRRSV: AMs inoculated with PCV2 first then inoculated with PRRSV 18 h later; PRRSV/PCV2: AMs inoculated with PRRSV first then inoculated with PCV2 18 h later; PCV2-PRRSV: AMs co-inoculated with PCV2 and PRRSV simultaneously. *Significantly different (*P* < 0.05) from the Mock at the same h post inoculation (HPI) with the first virus. ^a, b, c, d, e^Values with different labels at the same HPI differ significantly (P < 0.05).

The levels of IL-8 (Figure 4A) in all PCV2- and/or PRRSV-inoculated groups were 1390.0 ± 49.4 to 2035.5 ± 72.4 pg/ml and were all significantly higher (*P* < 0.001) than those (ranging from 299.2 ± 8.3 to 931.5 ± 21.3 pg/ml) in the Mock group. No statistically significant differences (*P* > 0.157) in the IL-8 levels in the supernatants of AMs were seen among all PCV2- and/or PRRSV-inoculated groups.

The levels of TNF-α (Figure 4B) were low in the supernatant of AMs in the Mock group during the experimental period. They ranged from 22.4 ± 5.5 to 55.1 ± 8.2 pg/ml. All PCV2- and/or PRRSV-inoculated groups showed a consistent and significant increase (*P* < 0.001) in the production of TNF-α in the supernatants of AMs than that in the Mock group during 18 to108 HPI. The levels of TNF-α in the supernatants of AMs in the PCV2 group were 188.8 ± 9.3 to 297.7 ± 5.3 pg/ml and were significantly lower (*P* < 0.028) than those in all PRRSV-inoculated groups throughout the experimental period. The levels of TNF-α in the supernatants of AMs in the PRRSV and PRRSV/PCV2 groups were 494.2 ± 25.1 to 878.4 ± 33.3 pg/ml and 832.4 ± 21.9 to 1149.2 ± 125.1 pg/ml, respectively, and were about fifteen- to twenty-fold higher than those of the Mock group. In the PCV2/PRRSV group, the levels of TNF-α in the supernatants of AMs were 446.9 ± 26.9 to 762.4 ± 20.1 pg/ml, which were significantly lower (*P* < 0.014) than those of the PRRSV group at 18 HPI and during 54 to 108 HPI, significantly lower (*P* < 0.003) than those of the PRRSV/PCV2 group during 18 to 108 HPI, and significantly lower (*P* < 0.012) than those of the PCV2-PRRSV group at 18, 54, and 90 HPI, respectively. The levels of TNF-α in the supernatants of AMs in the PCV2-PRRSV group were 253.9 ± 47.8 to 459.5 ± 27.9 pg/ml and significantly lower (*P* < 0.023)than those of the PRRSV group during 54 to 108 HPI and the PRRSV/PCV2 group during 18 to 108 HPI.

The levels of IFN-α bioactivity (Figure 4C) in the supernatants of AMs in the Mock and PRRSV groups were low throughout the experimental period. They ranged from 0 to 69.5 ± 21.5 U/ml and 75.6 ± 65.5 to 125.0 ± 20.3 U/ml, respectively. On the contrary, the levels of IFN-α in the supernatants of AMs increased from 1125.0 ± 425.7 to 4815.4 ± 221.5 U/ml in all PCV2-inoculated groups and were consistently and significantly higher (*P* < 0.001) than those of the Mock and PRRSV groups.

### Fas and FasL transcripts in PCV2- and/or PRRSV-inoculated swine AMs

**Figure 5:**
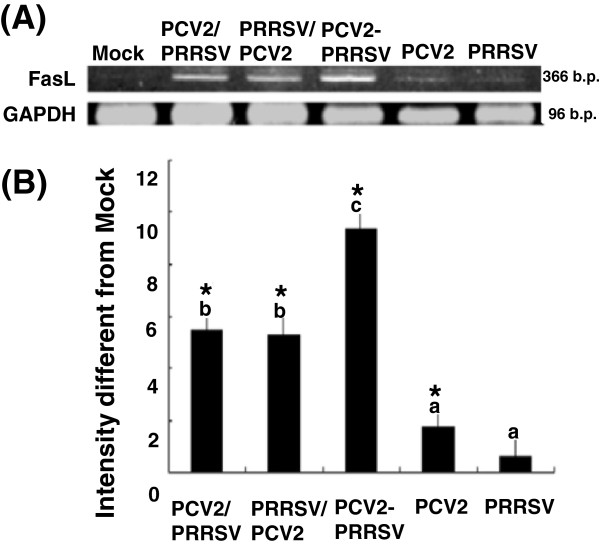
**Expression levels of FasL mRNA in PCV2- and/or PRRSV-inoculated swine alveolar macrophages (AMs).** The expression levels of FasL mRNA in all cultures were analyzed at 42 h after inoculation with the first virus by RT-PCR and electrophoresis. The sizes of FasL and GAPDH housekeeping gene are 366 and 96 b.p., respectively (**A**); values are further normalized using the housekeeping gene GAPDH and expressed as the relative intensity at the level different from that of mock-inoculated AMs (Mock) and shown as mean ± SD of three independent experiments (**B**). Mock: AMs inoculated with an equal volume of RPMI-C; PCV2: AMs inoculated with PCV2 alone; PRRSV: AMs inoculated with PRRSV alone; PCV2/PRRSV: AMs inoculated with PCV2 first then inoculated with PRRSV 18 h later; PRRSV/PCV2: AMs inoculated with PRRSV first then inoculated with PCV2 18 h later; PCV2-PRRSV: AMs co-inoculated with PCV2 and PRRSV simultaneously. *Significantly different (*P* < 0.05) from the Mock. ^a, b, c^Values with different labels differ significantly (P < 0.05).

After inoculation of AMs with one or both viruses or equal volume of RPMI-C at 42 HPI, the mRNA levels of Fas and FasL were evaluated. Neither AMs from the Mock group nor AMs from any PCV2- and/or PRRSV-inoculated groups expressed detectable level of Fas mRNA (data not shown); however, variable FasL mRNA levels were seen in all PCV2- and/or PRRSV-inoculated groups (Figure 5A, 5B). A significant increase (*P* < 0.001) in the FasL transcripts was demonstrated in AMs from all PCV2-inoculated groups as compared with that of the Mock group. All of the dually inoculated groups displayed a significant enhancement (*P* < 0.014) in the expression levels of FasL mRNA in AMs as compared with that of single virus inoculation. Among the PCV2 and PRRSV dually inoculated groups, the expression levels of FasL mRNA in AMs from the PCV2-PRRSV group was significantly higher (*P* < 0.016) than those of the PCV2/PRRSV and PRRSV/PCV2 groups.

## Discussion

A high prevalence of PCV2 and PRRSV co-infection is frequently observed in PRDC-affected pigs in many pig producing countries, including Taiwan [3,10,21,22]. The present study was conducted to study co-infection of PCV2 and PRRSV in swine AMs in the pathogenesis of PRDC by mimicking possible infection conditions/situations in the field [23,24]. Similar to our previous study [14], PCV2 was easily internalized in the cytoplasm of AMs but caused no noticeable cell death; and PRRSV displayed a low infectious rate but severe cytopathic effect and strong TNF-α induction in AMs. PCV2-induced IFN-α likely caused reduction in PRRSV infectious rate and PRRSV-related AMs dysfunction when AMs were co-inoculated with PCV2 and PRRSV simultaneously [14,18]. Similar PCV2-induced IFN-α effects were seen in the PCV2/PRRSV group but not in the PRRSV/PCV2 group, despite that a significant amount of IFN-α was also induced in PRRSV/PCV2 group, indicating that the pre-existing or co-existing PCV2 could interfere or hinder, at least partially, PRRSV infection. Our previous [14,18] and present studies have demonstrated that a significant amount of PRRSV antigens and PRRSV-induced cell death and dysfunctions in AMs could be detected within the first 18 HPI when inoculation of AMs with PRRSV. Those findings indicate that PRRSV replication and PRRSV-induced dysfunction and cytokine production in AMs should have occurred during the first 18 HPI after inoculation of AMs with PRRSV. Thus, it is reasonable to suggest that as long as PRRSV has established its infection in AMs prior to PCV2 inoculation or infection, the subsequent IFN-α production induced by infection with PCV2 later is incapable of curtailing those adverse effects caused by previous or pre-existing PRRSV infection.

In the study reported by Buddaert et al. [25], pigs infected with porcine respiratory coronavirus virus (PRCV) 2 days prior to infection with PRRSV showed a reduction in PRRSV titer but had no change in PRCV-induced IFN-α production. Similarly, no interference with PCV2-induced IFN-α production was seen in all groups with dual infection of PCV2 and PRRSV in the present study. On the contrary, the study of Albina et al. [26] showed that swine AMs pre-infected with PRRSV 6 h prior to the inoculation of swine transmissible gastroenteritis virus (TGEV) *in vitro* resulted in a complete inhibition in TGEV-induced IFN-α production. The above mentioned results indicate that the interactions among different viruses are complicated and may lead to different disease courses in the field.

Simultaneous infection of PCV2 and PRRSV is frequently encountered in pig herds worldwide [10,27]. Various field and *in vivo* and *in vitro* experimental studies have been conducted to evaluate the effects of co-infection of PCV2 and PRRSV [9,10,12-14,27,28]. Simultaneous inoculation with PRRSV and PCV2 viruses [9,10], or PRRSV inoculated one week prior to PCV2 [28], was commonly used in those *in vivo* studies. Results from these field and experimental studies have demonstrated that PRRSV could cause enhanced PCV2 replication evidenced by higher serum and tissue PCV2 loads, increased severity of the pathological changes and clinical manifestation of PCVAD, and higher incidence of PCVAD [9,10,27,28]. Our previous study has demonstrated that PCV2 cannot efficiently replicate in AMs unless being activated such as by lipopolysaccharide *in vitro*[29], and this may explain why, in contrast to the above mentioned *in vivo* studies, no significant changes in PCV2 antigen-positive rate were seen in the present study. Owing to that no *in vivo* study with PCV2 inoculation first followed by PRRSV has been reported, it would be of interest to see whether those alterations seen in PCV2/PRRSV group in the present study can be reproduced *in vivo*.

Similar to the findings of our previous studies [15,18], transient decrease in phagocytosis but persistent reduction in microbicidal capability in the group inoculated with PCV2 alone and constant decrease in phagocytosis and microbicidal capability in all PRRSV-inoculated groups were noted in the present study. The significantly higher PRs of AMs in the PCV2/PRRSV and PCV2-PRRSV groups than those in the PRRSV group during 90 to 108 HPI indicate that PCV2 that was inoculated first or simultaneously not only hinder PRRSV replication but also reduce the PRRSV-induced adverse effects on the phagocytosis of swine AMs. Although with some variations, the constant and significant reduction in the capability of swine AMs to kill *C. albicans* in all PCV2- and/or PRRSV-inoculated groups suggests that PCV2 and/or PRRSV infection may lead to the survival and proliferation of the opportunistic or secondary pathogens and lesion development in pig lungs. This is in agreement with the findings in PRDC-affected pigs in the field [4,30]. As suggested by the previous studies [15,18], the impaired microbicidal capability in PCV2- and/or PRRSV-inoculated groups may be partially due to the reduction in reactive oxygen species production of swine AMs.

PCV2 can be further subdivided into two main subtypes, PCV2a and PCV2b [31]. Although PCV2a has been considered as less virulent compared to PCV2b based on field observation [32], no experimental studies have been able to support this speculation by using PCV2 infection alone [33] or PCV2 and PRRSV co-infection [34]. Our preliminary study with PCV2a also showed no difference from PCV2b on the reduction of the phagocytotic and microbial killing capabilities of AMs *in vitro* (unpublished data).

Fas (CD95)/Fas ligand (FasL) has been shown to play a major role in the induction of apoptosis in immune cells and bronchial epithelial cells [35-37] and mediation of neutrophil chemotaxis [38-40]. The ability of FasL to induce acute inflammatory response is even stronger than that of bacterial lipopolysaccharide (LPS) [41]. The FasL expressed by AMs has also been suggested to participate in the pathogenesis of acute respiratory disease syndrome (ARDS) [41]. To further elucidate the possible mechanism of pulmonary inflammatory response and tissue injury during PCV2 and/or PRRSV infection, Fas and FasL expressions were also analyzed in the present study. The results of FasL mRNA expression clearly showed that PCV2 but not PRRSV could stimulate swine AMs to produce FasL; however, PRRSV had the addictive effect on PCV2-related FasL mRNA expression in swine AMs when PRRSV co-existed with PCV2. Comparing with PCV2/PRRSV or PRRSV/PCV2 group, a significantly higher level of FasL mRNA expression was seen in the PCV2-PRRSV group. At 42 HPI, the duration for co-existence of PCV2 and PRRSV in the PCV2/PRRSV or PRRSV/PCV2 group was 18 h less than that in the PCV2-PRRSVgroup. The result suggests that the addictive effect of PRRSV on the enhancement of FasL mRNA expression by PCV2 corresponds with the duration for co-existence and interaction of PCV2 and PRRSV. A significant increase in FasL mRNA expression with no detectable Fas mRNA expression in all PCV2- and/or PRRSV- inoculated AMs suggests that Fas/FasL may not be directly involved in the apoptosis and other cytopathologies in the PCV2- and/or PRRSV-inoculated AMs.

Pigs naturally infected with PCV2 and/or PRRSV frequently show interstitial pneumonia [10,42]. Inoculation of PCV2 or PRRSV alone has been shown to cause mild to moderate interstitial pneumonia in conventional pigs; however, severe interstitial pneumonia with occasional peribronchiolar mononuclear cell cuffing and scattered individual bronchiolar epithelial cellular necrosis could be induced in Cesarean-derived colostrum-deprived pigs with dual infection of PCV2 and PRRSV [9,30]. Interferon-α activation in cellular immunity has been suggested to complicate interstitial pneumonia [37]. Tumor necrosis factor-α can induce strong proinflammatory response, including the release of inflammatory mediators and chemokines [43]. Interleukin 8 is an acute inflammatory chemokine for neutrophils and also considered as a key factor in the pathogenesis of interstitial pneumonia [44]. In addition, FasL has been suggested to be associated with the process of pulmonary inflammation and vascular permeability and the induction of epithelial apoptosis when it ligates with the airway epithelial cells expressing Fas [39,41]. Taken together, the increased expression of IFN-α, TNF-α, IL-8, and FasL mRNA in AMs from pigs in various infection orders by PCV2 and PRRSV observed in the present study, to some extent may contribute to pneumonia and bronchiolar epithelial damage in the lungs of PCV2- and/or PRRSV-infected pigs.

## Competing interests

The authors declare that they have no competing interests.

## Authors’ contributions

All authors conceived of this study and participated in its design. YCT, HWC, TLL, CHW, and VFP drafted the manuscript. HWC and YCT collected data and performed statistical analysis with the help by CML. CRJ, TLL, CHW, and VFP provided help with the interpretation of results. All authors read and approved the final manuscript.
